# Pachydermoperiostosis mimicking the acral abnormalities of acromegaly

**DOI:** 10.1007/s12020-019-02168-5

**Published:** 2020-01-08

**Authors:** Pedro Marques, Maria Stelmachowska-Banas, David Collier, Florian Wernig, Márta Korbonits

**Affiliations:** 1 Centre for Endocrinology, William Harvey Research Institute, Barts and the London School of Medicine and Dentistry, Queen Mary University of London, London, EC1M 6BQ UK; 2 Department of Endocrinology, Centre of Postgraduate Medical Education, Warsaw, 01-809 Poland; 3 Endocrinology, Imperial College Healthcare NHS Trust, London, W6 8RF UK

Bones and soft tissues of hands and feet can be affected by different conditions, including genetic, metabolic or systemic disorders [1]. Acromegaly is a condition caused by excessive secretion of growth hormone (GH) leading to elevated insulin growth factor-1 (IGF-1) levels, which is characterised by somatic overgrowth and physical disfigurement notably affecting hands and feet [2]. However, there are other conditions that can mimick the clinical features seen in acromegaly without GH/IGF-1 anomalies, termed as pseudoacromegaly [1].

**Fig. 1:**
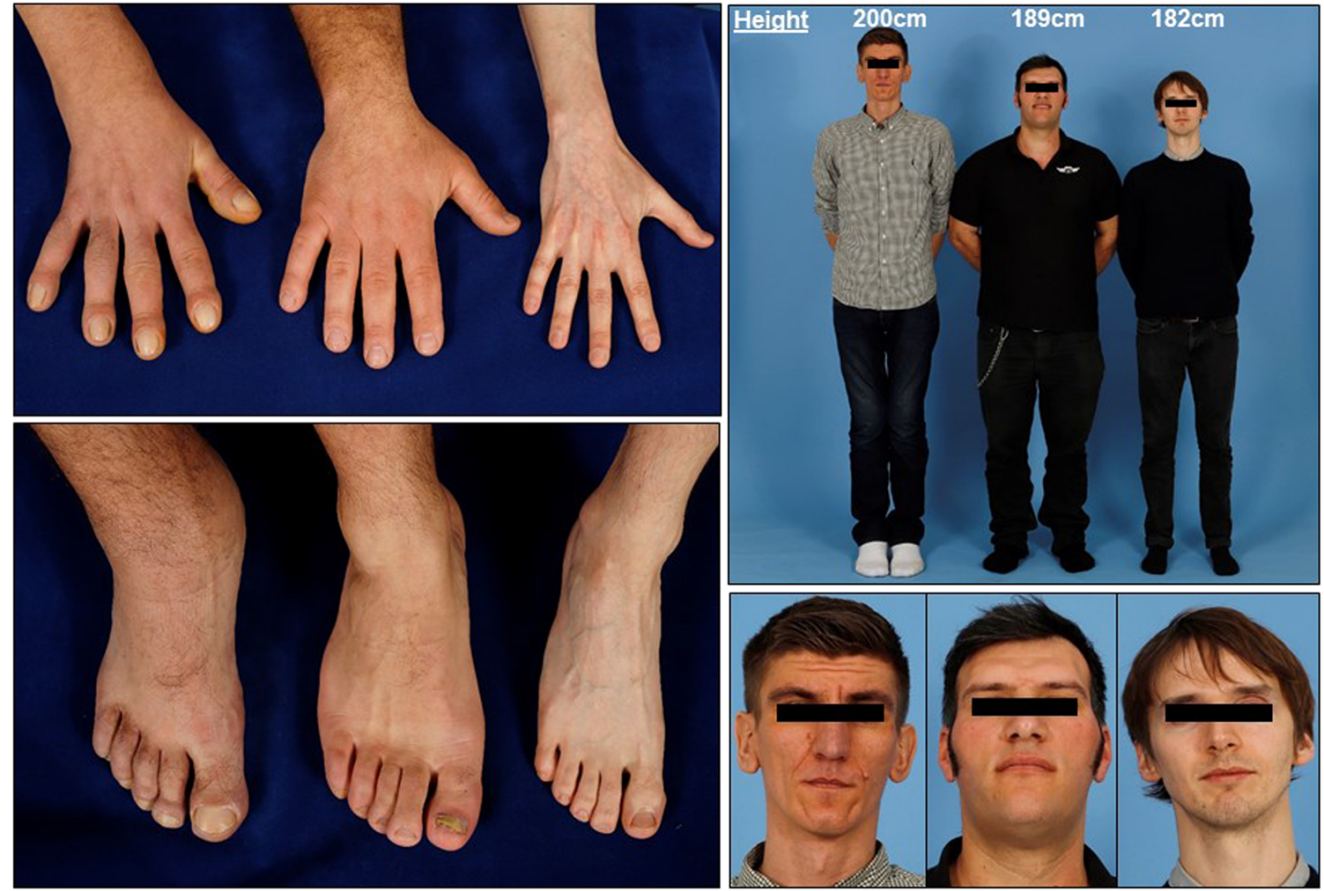
Hands, feet and facial appearance of a tall patient with pachydermoperiostosis (left subject), in comparison with a patient with acromegaly (middle subject) and a healthy male (right subject). Note the digital clubbing and the loss of contour of the wrist and ankle in the patient with pachydermoperiostosis

A 3-year-old boy was noted to have large hands and feet, severe joint pain and hyperhidrosis. Later he developed forehead skin furrowing (pachydermia), but was not diagnosed until the age of 26, when he was referred to an endocrinologist with the suspicion of acrogigantism. IGF-1 level was not elevated, random GH 0.14 µg/l, GH after glucose-load was 0.46 µg/l. He has pachydermoperiostosis due to a homozygous *HPGD* (15-hydroxyprostaglandin-dehydrogenase) mutation (c.175-176delCT). His hands and feet are remarkably large and fleshy with long and thickened fingers and toes, prominent digital clubbing and loss of the normal contour of the widened wrists and ankles (Fig. 1, left subject). Similar acral features can be seen in acromegaly (Fig. 1, middle subject), while both are remarkably different from a healthy male (Fig. 1, right subject). The left patient had other clinical features characteristic of pachydermoperiostosis, including seborrhoea, long eyelashes, blepharoptosis, peri-articular oedemas, synovial effusions and periodic watery diarrhoea. Although tall stature is not a classical feature of pachydermoperiostosis [3], this patient’s height (200 cm, midparental-height 181 cm) adds further diagnostic challenges to distinguish from young-onset acromegaly. His brother presented at the age of 3 with arthralgia and bone and skin manifestations; he carries the same homozygous mutation, while parents are heterozygous.

Pachydermoperiostosis (primary autosomal recessive hypertrophic osteoarthropathy) is a rare condition characterised by digital clubbing, joint problems and pachydermia, but other skin manifestations due to dermal and sebaceous gland hypertrophy can be found [1]. HPGD loss leads to accumulation of circulating prostaglandins, suggested to play a role in the development of the characteristic signs and symptoms [3]. Pachydermoperiostosis-related acral abnormalities may overlap with those seen in acromegaly, including broadened extremities, widened, thickened and stubby fingers, and thickened soft tissue. In acromegaly, excessive GH/IGF-1 leads to periosteal bone formation, growth of synovial tissue, cartilage and leading to hypertrophic arthropathy associated with pain and deformity [2], as also seen in pachydermoperiostosis. Cutis verticis gyrate, facial coarsening, hyperhidrosis, seborrhoea and acne are common in pachydermoperiostosis as well as in acromegaly. However, other manifestations characteristic in pachydermoperiostosis, but not seen in acromegaly, are blepharoptosis, long eyelashes, myelofibrosis, hypoalbuminemia, peptic ulcer, gastric cancer or watery diarrhoea in response to certain triggers, such as cold drinks, greasy food or sexual activity [1]. Pachydermoperiostosis diagnosis is often established by geneticists or rheumatologists; however, these patients may be first referred for investigation of possible GH excess to an endocrinologist, as in our case and in several cases reported in the literature, thus we should be aware of pachydermoperiostosis as a pseudoacromegaly condition and aid in establishing its diagnosis [1].
